# The ZMAGE-A3 affitoxin 172-cpp has great potential for targeting therapy of tumors

**DOI:** 10.3389/fonc.2026.1862676

**Published:** 2026-10-28

**Authors:** Hongjing Li, Kui Chen, Li Shi, Yan-Ru Guo, Dong-Yin Xu, Xiang-Tao Zheng, Li-Fang Zhang

**Affiliations:** 1 Department of Urology II, Handan First Hospital, Handan, Hebei, China; 2 Department of Microbiology and Immunology, Institute of Molecular Virology and Immunology, School of Basic Medical Sciences, Wenzhou Medical University, Wenzhou, Zhejiang, China; 3 Department of Vascular Surgery, The Second Affiliated Hospital and Yuying Children’s Hospital of Wenzhou Medical University, Wenzhou, Zhejiang, China; 4 Department of Orthopaedics, The Second Affiliated Hospital and Yuying Children’s Hospital of Wenzhou Medical University, Wenzhou, Zhejiang, China

**Keywords:** affitoxin, MAGE-A3, melanoma, molecular imaging, targeted therapy

## Abstract

**Background:**

The MAGE-A3 oncological antigen, a member of the cancer-testis protein family, is markedly overexpressed across various types of malignant tumors. Affibody molecules, a novel class of compact targeting proteins (~6.5 kDa), have been established as potent agents for molecular imaging and targeted cancer therapy. This study evaluates the designed an affibody molecules as novel agents by fusing a MAGE-A3 affibody molecule with modified *Pseudomonas* exotoxin (PE38KDEL) and a cell-penetrating peptide (cpp) to develop an affitoxin, ZMAGE-A3 affitoxin 172-cpp, and evaluated its anti-tumor efficacy.

**Methods:**

we verified the expression of ZMAGE-A3 affitoxin 172-cpp by SDS-PAGE. MAGE-A3-positive cells was selected by qPCR. The internalization of ZMAGE-A3 affitoxin 172-cpp was detected using fluorescence imaging. Cell viability was detected using cell counting kit 8 (CCK-8) and colony formation assay. Cell apoptosis was measured by flow cytometry. The *in vivo* targeting ability of ZMAGE-A3 affitoxin 172-cpp was analyzed by near-infrared (NIR) imaging.

**Results:**

ZMAGE-A3 affitoxin 172-cpp demonstrated precise binding to the MAGE-A3 protein in living cells. Moreover, the accumulation of DyLight755-labeled ZMAGE-A3 affitoxin 172-cpp in MAGE-A3-positive tumors was observed as early as 1 hour post-injection, with clearance by 48 hours. ZMAGE-A3 affitoxin 172-cpp effectively suppressed the viability and induced apoptosis of A375 and A2058 cells. The results from xenograft model indicated that ZMAGE-A3 affitoxin 172-cpp effectively repressed *in vivo* tumor growth.

**Conclusion:**

Our findings support the potential of the ZMAGE-A3 affitoxin 172-cpp as a molecular imaging and therapeutic agent for MAGE-A3 positive cancer.

## Introduction

Melanoma-associated antigen A3 (MAGE-A3) is a member of the cancer-testis antigen family, which is typically expressed only in the testis of healthy adults but frequently overexpressed in a variety of malignant tumors, including melanoma, breast, lung, colorectal, esophageal, multiple myeloma, and gastric cancers (1–4). The restricted expression in normal tissues and high immunogenicity in tumors make MAGE-A3 an attractive target for cancer diagnosis and immunotherapy (5, 6). Therapies employing CTLA-4 checkpoint inhibitors have been shown to reduce MAGE-A3 mRNA levels (7), and MAGE-A3 overexpression has been associated with resistance to EGFR tyrosine kinase inhibitors and chemotherapy (8). Therefore, developing MAGE-A3-targeted agents holds promise for both tumor imaging and targeted therapy.

Monoclonal antibodies have been successfully used for targeted cancer therapy; however, their large molecular weight (~150 kDa) limits tumor penetration and results in slow blood clearance, which can lead to suboptimal imaging contrast (9). Affibody molecules are small (approximately 6.5 kDa) engineered scaffold proteins derived from the B domain of staphylococcal protein A. They exhibit high affinity and specificity for their targets, similar to antibodies, but offer several advantages, including low immunogenicity, rapid renal clearance, excellent tissue penetration, and high physicochemical stability (10). Over the past decades, affibodies targeting HER2, EGFR, and HPV16/18 E7 have been developed for molecular imaging and therapy (11). Notably, the HER2-targeting affibody ZHER2:342 has been successfully used in clinical imaging of breast cancer (12). These properties make affibody molecules promising candidates for *in vivo* molecular imaging and targeted therapy.

To convert the targeting ability of affibodies into potent therapeutic effects, toxin moieties can be conjugated to affibodies to generate “affitoxins”. In this study, we fused a MAGE-A3-specific affibody (ZMAGE-A3 172) with a modified Pseudomonas exotoxin (PE38KDEL) and a cell-penetrating peptide (cpp). The PE38KDEL toxin has been engineered to retain in the endoplasmic reticulum by interacting with the KDEL receptor, thereby enhancing its cytotoxicity through ADP-ribosylation of elongation factor 2 (EF2) and subsequent induction of apoptosis (13). The incorporation of cpp facilitates cellular internalization by overcoming the hydrophobic barrier of cell membranes, which is expected to augment the biological activity of the affitoxin (14). To date, no affitoxin targeting MAGE-A3 has been reported.

In the present study, we constructed a novel affitoxin, designated ZMAGE-A3 affitoxin 172-cpp, and evaluated its targeting specificity, cellular internalization, anti-tumor activity *in vitro* and *in vivo*, as well as its potential for near-infrared imaging in xenograft mouse models. Our findings demonstrate that ZMAGE-A3 affitoxin 172-cpp is a promising dual-function agent for molecular imaging and targeted therapy of MAGE-A3-positive cancers.

## Materials and methods

### Cell culture

The human melanoma cell line A375 and human skin cancer cell line A2058 were procured from the Shanghai Cell Repository of the Chinese Academy of Sciences. A431 cells (cutaneous squamous cell carcinoma) were purchased from the American Type Culture Collection (ATCC, Manassas, VA, USA). All cells were cultured in Dulbecco’s Modified Eagle Medium (DMEM, high glucose, Gibco, Grand Island, NY, USA) supplemented with 10% fetal bovine serum (FBS, Gibco) and 1% penicillin–streptomycin (Beyotime, Shanghai, China) at 37 °C in a humidified atmosphere containing 5% CO_2_. Cells were passaged at 70%–80% confluence.

### Affibody expression, purification and western blot confirmation

The DNA sequences of wild-type Z_WT_ control affibody, Z_MAGE-A3_ 172 (targeting MAGE-A3), and Z_MAGE-A3_ affitoxin 172-cpp (MAGE-A3 affibody fused to modified Pseudomonas exotoxin PE38KDEL and a cell-penetrating peptide) were synthesized (GenScript, Nanjing, China) and cloned into the pET21a(+) expression vector (Novagen, Madison, WI, USA). The recombinant vectors were confirmed by DNA sequencing. Recombinant proteins were expressed in *Escherichia coli* BL21(DE3) cells (TransGen Biotech, Beijing, China). Protein expression was induced by adding 0.8 mM isopropyl β-D-1-thiogalactopyranoside (IPTG, Beyotime, Shanghai, China) when the culture reached an OD_600_ of 0.6–0.8, followed by incubation at 37 °C for 4 h with shaking at 250 rpm. Bacterial pellets were harvested by centrifugation (6, 000 × g, 15 min, 4 °C), resuspended in lysis buffer (50 mM NaH_2_PO_4_, 300 mM NaCl, 10 mM imidazole, pH 8.0), and lysed by sonication on ice (Scientz-IID, Ningbo Scientz Biotechnology Co., Ltd., Ningbo, China). The lysate was clarified by centrifugation (12, 000 × g, 30 min, 4 °C). Recombinant His-tagged affibody proteins were purified using Ni-NTA Sepharose affinity chromatography (Qiagen, Hilden, Germany) according to the manufacturer’s protocol, followed by dialysis against PBS (pH 7.4). Protein purity was confirmed by 12% SDS-PAGE (Bio-Rad Laboratories, Hercules, CA, USA) with Coomassie Brilliant Blue R-250 staining. For Western blot, purified proteins were separated by 12% SDS-PAGE, transferred onto polyvinylidene difluoride membranes (Millipore, Billerica, MA, USA), blocked with 5% non-fat milk for 1 h at room temperature, and incubated overnight at 4 °C with mouse anti-His-tag monoclonal antibody (1:2, 000, Beyotime). After three washes with TBST, membranes were incubated with HRP-conjugated goat anti-mouse IgG secondary antibody (1:5, 000, Beyotime) for 1 h at room temperature, and visualized using enhanced chemiluminescence (ECL, Beyotime).

### qPCR assay for MAGE-A3 expression

Total RNA was extracted from A375, A2058 and A431 cells using TRIzol reagent (Invitrogen, Carlsbad, CA, USA) following the manufacturer’s protocol. RNA concentration and purity were assessed with a NanoDrop 2000 spectrophotometer (Thermo Fisher Scientific, Waltham, MA, USA). Complementary DNA (cDNA) was synthesized using the PrimeScript™ RT Reagent Kit with gDNA Eraser (Takara Bio Inc., Kusatsu, Japan) according to the manufacturer’s instructions. Quantitative real-time PCR (qPCR) was performed on a 7300 Applied Biosystems Real-Time PCR System using TB Green^®^ Premix Ex Taq™ II (Takara). Thermocycling conditions were: 95 °C for 30 s, followed by 40 cycles of 95 °C for 5 s and 60 °C for 34 s. The primer sequences used were: MAGE-A3 forward, 5′-GGGACGGACATCTTCACTGG-3′; MAGE-A3 reverse, 5′-CCACCAATTCTTGTGGCACC-3′; GAPDH forward, 5′-GCACCGTCAAGGCTGAGAAC-3′; GAPDH reverse, 5′-TGGTGAAGACGCCAGTGGA-3′. Relative MAGE-A3 mRNA expression levels were calculated using the 2^-ΔΔCt^ method and normalized to GAPDH. A431 cells were used as negative control because they express minimal MAGE-A3 [4†L24-L28].

### Immunofluorescence imaging

A375, A2058 and A431 cells were seeded on sterilized glass coverslips in 24-well plates at 2 × 10^4^ cells per well and cultured overnight to allow adherence. Cells were fixed with 4% paraformaldehyde (Beyotime) for 15 min at room temperature, permeabilized with 0.1% Triton X-100 (Sigma-Aldrich, St. Louis, MO, USA) for 10 min, and blocked with 5% BSA (Bovogen, Melbourne, Australia) for 1 h. Cells were then incubated with 2 μM Z_MAGE-A3–_172 or Z_MAGE-A3_ affitoxin 172-cpp overnight at 4 °C. After three washes with PBS, cells were incubated with DyLight™ 594-conjugated goat anti-mouse IgG (1:200, Thermo Fisher Scientific) for 1 h at room temperature in the dark. Nuclei were counterstained with Hoechst 33342 (5 μg/mL, Beyotime) for 10 min. Coverslips were mounted on slides with anti-fade mounting medium (Beyotime). Fluorescence images were acquired using a Leica TCS SP8 confocal laser scanning microscope (Leica Microsystems, Wetzlar, Germany). For quantitative analysis of intracellular fluorescence intensity, images were processed using ImageJ software (NIH, Bethesda, MD, USA). The integrated fluorescence density of at least 50 randomly selected cells per group was measured and normalized to cell number.

### Colony formation assay

For the colony formation assay, A375 and A2058 cells were seeded in 6-well plates at 1 × 10³ cells per well and allowed to attach overnight. Cells were then treated with one of the following agents for 48 h: Z_WT_ control (2 μM), Z_MAGE-A3_ 172 (2 μM), or Z_MAGE-A3_ affitoxin 172-cpp (2 μM). After treatment, cells were trypsinized, washed with PBS, and resuspended in fresh culture medium. The treated cells (1 × 10³ cells per well) were mixed with 1.5 mL of 0.3% top agar (Beyotime) and plated onto a pre-solidified 0.6% base agar layer in 6-well plates. Plates were incubated at 37 °C in a 5% CO_2_ incubator for 14 days, with fresh medium added every 3–4 days. After incubation, colonies were fixed with 4% paraformaldehyde for 15 min at room temperature and stained with 0.5% crystal violet solution (w/v, prepared in 20% methanol) for 30 min at room temperature (8). Excess stain was removed by gently washing the plates with tap water. Colonies (≥50 cells per colony) were counted under a dissection microscope (Olympus SZX7, Tokyo, Japan). All assays were performed in triplicate.

### Cell counting kit-8 viability assay

Cell viability was evaluated using the CCK-8 assay (Beyotime). A375 and A2058 cells were seeded in 96-well plates at 5 × 10³ cells per well in 100 μL of culture medium and incubated overnight at 37 °C. The medium was then replaced with fresh medium containing Z_WT_ control (2 μM), Z_MAGE-A3_ 172 (2 μM), or Z_MAGE-A3_ affitoxin 172-cpp (2 μM), with untreated wells serving as negative controls. After 48 h of treatment, 10 μL of CCK-8 solution was added to each well, and the plates were incubated for an additional 2 h at 37 °C in a humidified incubator. Absorbance at 450 nm was measured using a Spark^®^ multimode microplate reader (Tecan Group Ltd., Männedorf, Switzerland). All experiments were performed in five independent replicates (n = 5), each with three technical replicates. Viability was expressed as percentage relative to untreated control cells, which were set to 100%.

### Apoptosis assay by flow cytometry

Apoptosis was assessed using the Annexin V-FITC/PI Apoptosis Detection Kit (Beyotime). A375 and A2058 cells were seeded in 6-well plates at 2 × 10^5^ cells per well and cultured overnight. Cells were then treated with Z_WT_ control (2 μM), Z_MAGE-A3_ 172 (2 μM), or Z_MAGE-A3_ affitoxin 172-cpp (2 μM) for 48 h. Untreated cells served as controls. After treatment, cells were harvested by trypsinization (0.25% trypsin-EDTA, Gibco), washed twice with cold PBS, and resuspended in 195 μL of 1× binding buffer. Cells were then stained with 5 μL of Annexin V-FITC and 5 μL of propidium iodide (PI) for 15 min at room temperature in the dark. Stained cells were analyzed within 1 h using a BD FACSCanto™ II flow cytometer (BD Biosciences, San Jose, CA, USA). Early apoptotic cells (Annexin V^+^/PI^-^), late apoptotic cells (Annexin V^+^/PI^+^), and total apoptotic cells (sum of early and late) were quantified using FlowJo software (v10.8, BD Biosciences). All experiments were performed in triplicate.

### *In vivo* near-infrared imaging

The *in vivo* tumor-targeting ability of Z_MAGE-A3_ affitoxin 172-cpp was assessed in female BALB/c nude mice (6–7 weeks old, 18–20 g) purchased from Shanghai SLAC Laboratory Animal Co., Ltd. (Shanghai, China). All animal procedures were approved by the Animal Ethics Committee of The Second Affiliated Hospital and Yuying Children’s Hospital of Wenzhou Medical University (Approval No. LAC-2023-04-12A), and were conducted in strict accordance with the National Institutes of Health Guide for the Care and Use of Laboratory Animals (8th edition, 2011).

To establish xenograft models, A375 and A2058 cells (2 × 10^6^ cells in 100 μL PBS) were injected subcutaneously into the right forelimb of each mouse. When tumors reached a volume of 300–500 mm³ (approximately 3 weeks after implantation), mice were randomized into experimental groups (n = 5 per group). Z_WT_, Z_MAGE-A3_ 172, and Z_MAGE-A3_ affitoxin 172-cpp were conjugated with the near-infrared fluorescent dye DyLight™ 755 NHS Ester (Thermo Fisher Scientific) according to the manufacturer’s instructions. For imaging, mice were anesthetized with 1.4% isoflurane (RWD Life Science, Shenzhen, China) in oxygen. Mice were then intravenously injected via the tail vein with 100 μL of PBS containing 100 μmol of DyLight755-labeled affibody molecules. Fluorescence images were acquired using a Maestro™ 2.10 *in vivo* imaging system (Caliper Life Sciences, Hopkinton, MA, USA) at multiple time points post-injection (1 h, 2 h, 4 h, 6 h, 8 h, 12 h, 24 h, 48 h, and 72 h). After the final time point, mice were euthanized by cervical dislocation under deep isoflurane anesthesia, and tumors and major organs (heart, liver, spleen, lung, kidney, skin) were excised for *ex vivo* imaging.

### Xenograft tumor model and therapeutic evaluation

For therapeutic efficacy evaluation, A375 or A2058 cells (2 × 10^6^ cells in 100 μL PBS) were injected subcutaneously into the right forelimb of female BALB/c nude mice (n = 5 per group). When tumors reached approximately 10 mm³ (visible as palpable nodules, typically 5–7 days after implantation), mice were randomly assigned to one of four treatment groups: (1) PBS vehicle control, (2) Z_MAGE-A3_ 172 (100 μmol in 150 μL PBS), (3) Z_MAGE-A3_ affitoxin 172-cpp (100 μmol in 150 μL PBS), or (4) anti-MAGE-A3 antibody (100 μg in 150 μL PBS, Abcam, Cambridge, MA, USA). Treatments were administered via intravenous tail vein injection once every three days for a total of three injections (days 1, 4, 7). Tumor dimensions were measured every three days using a digital caliper, and tumor volume was calculated using the formula: volume (mm³) = (length × width²)/2. Body weights were recorded at each time point. After 21 days, mice were euthanized as described above, and tumors were excised, photographed, and weighed. The animal study protocol was approved by the Animal Ethics Committee of The Second Affiliated Hospital and Yuying Children’s Hospital of Wenzhou Medical University (Approval No. LAC-2023-04-12A).

### Statistical analysis

Data are presented as mean ± standard deviation (SD) from at least three independent experiments. Normality was assessed using the Shapiro-Wilk test, and homogeneity of variances was confirmed by Levene’s test. For comparisons involving three or more independent groups, one-way analysis of variance (ANOVA) followed by Tukey’s honestly significant difference (HSD) post-hoc test was used for pairwise comparisons between groups. Comparisons between two groups were performed using two-tailed Student’s t-test. A P-value < 0.05 was considered statistically significant. Statistical significance levels were annotated in the figures as follows: *P < 0.05, **P < 0.01, ***P < 0.001, ns = not significant. All statistical analyses were carried out using SPSS software (version 26.0, IBM Corp., Armonk, NY, USA) and GraphPad Prism 9.0 (GraphPad Software, La Jolla, CA, USA).

## Results

### Expression and identification of Z_MAGE-A3_ affitoxin 172-cpp

We first fused this Z_MAGE-A3–_172 molecule with modified Pseudomonas Exotoxin (PE38KDEL) and a cell-penetrating peptide (cpp) to develop an affitoxin, Z_MAGE-A3_ affitoxin 172-cpp (short as affitoxin). The results from the SDS-PAGE identified that purified affibody molecules had a molecular weight of around 7.5 kDa (**Figure 1A**). Through the western blot experiment, we observed that the Z_MAGE-A_ 172 and affitoxin fusion proteins reacted specifically with anti-His-tag mouse polyclonal antibodies (**Figure 1B**). (**Figure 1C**) Schematic representation of Z_MAGE-A3_ affitoxin 172-cpp showing the MAGE-A3-targeting affibody domain (Z_MAGE-A3_ 172), the PE38KDEL toxin domain, the cell-penetrating peptide (cpp), and the C-terminal His-tag.

**Figure 1:**
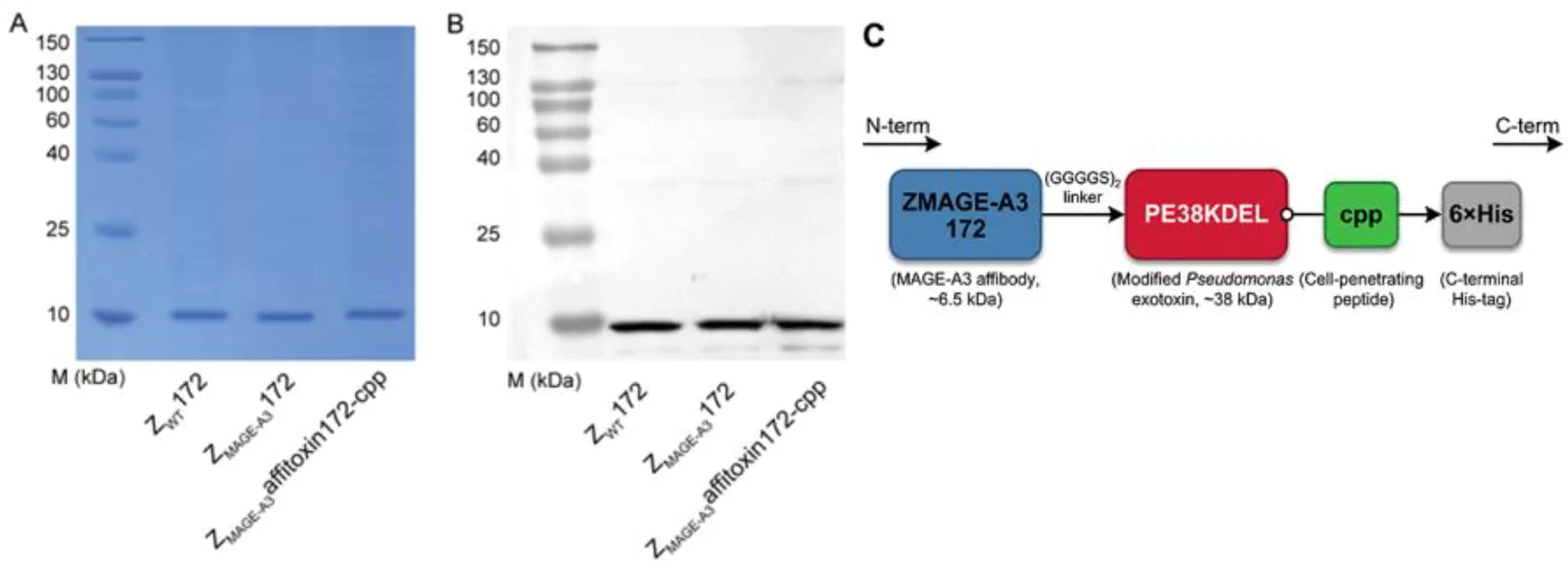
Expression and identification of Z_MAGE-A3_ affitoxin 172-cpp. **(A)** The expression of Z_WT_ 172, Z_MAGE-A3_ 172, and Z_MAGE-A3_ affitoxin 172-cpp visualized by SDS-PAGE. **(B)** The expression of Z_WT_ 172, Z_MAGE-A3_ 172, and Z_MAGE-A3_ affitoxin 172-cpp visualized by using anti-his antibody. **(C)** Schematic representation of Z_MAGE-A3_ affitoxin 172-cpp showing the MAGE-A3-targeting affibody domain (Z_MAGE-A3_ 172), the PE38KDEL toxin domain, the cell-penetrating peptide (cpp), and the C-terminal His-tag.

### Highly specific interaction of Z_MAGE-A3_ affitoxin 172-cpp with native MAGE-A3 *in vitro*

To determine the effects of Z_MAGE-A3_ affitoxin 172-cpp on cancer cells, we measured the expression of MAGE-A3 in A375, _A2058_ and A431 cells using qPCR and western blotting. The findings disclosed the presence of MAGE-A3 mRNA and protein in the A375 and _A2058_ cell lines, as depicted in **Figures 2A, B**. Subsequently, we employed an indirect immunofluorescence assay to examine the interaction between Z_MAGE-A3_ affibody molecules and the native MAGE-A3 protein in MAGE-A3-positive cells. Z_MAGE-A3_ and Z_wt_ were incubated with three distinct cell lines, and the findings indicated that only the MAGE-A3-positive cells (A375 and _A2058_) exhibited red fluorescence, signifying the binding of the affibody molecules within the cells (**Figures 2C, D**). Moreover, the fluorescence was predominantly localized to the cytoplasm and the perinuclear region (**Figures 2C, D**). Quantitative analysis of red fluorescence intensity using ImageJ (normalized to cell number) revealed a significant increase in A375 and A2058 cells treated with Z_MAGE-A3–_172 or Z_MAGE-A3_ affitoxin 172-cpp compared with A431 cells (P < 0.001, one-way ANOVA; **Figure 2E**).

**Figure 2:**
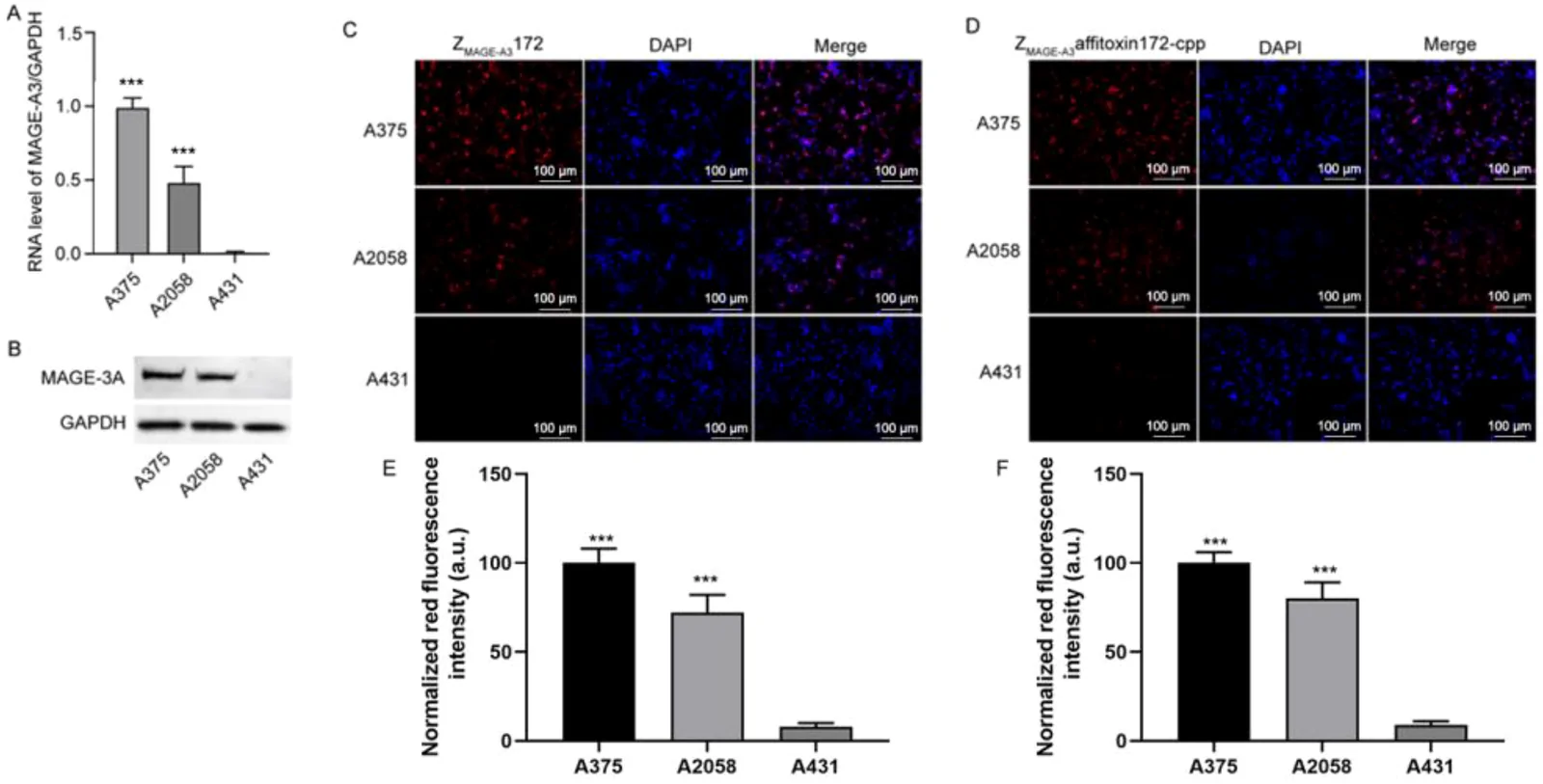
Highly specific interaction of ZMAGE-A3 with native MAGE-A3 *in vitro*. **(A)** qPCR analysis of MAGE-A3 mRNA expression in A375, A2058 and A431 cells. **(B)** Western blotting of MAGE-A3 protein expression in the same cell lines. **(C)** Immunofluorescence images of A375, A2058 and A431 cells incubated with Z_MAGE-A3_ 172. **(D)** Immunofluorescence images of the same cell lines incubated with Z_MAGE-A3_ affitoxin 172-cpp. Red fluorescence indicates binding of the affibody or affitoxin; nuclei were counterstained with Hoechst 33342 (blue). Scale bars: 100 µm. **(E)** Quantitative analysis of red fluorescence intensity normalized to cell number, measured using ImageJ from three independent experiments (n = 3 per group). Data are presented as mean ± SD. Statistical significance was determined by one-way ANOVA followed by Tukey’s HSD *post-hoc* test. ***P < 0.001 compared to A431+affitoxin control.

### The Z_MAGE-A3_ affitoxin 172-cpp targets tumor cell proliferation *in vitro*

Z_MAGE-A3_ affitoxin 172-cpp and Z_MAGE-A3–_172 both notably suppressed the viability (**Figures 3A, B**) and colony formation ability (**Figures 3C, D**) of A375 and A2058 cells, among which Z_MAGE-A3_ affitoxin 172-cpp exhibited stronger anti-proliferation ability. Cell apoptosis was also notably induced by Z_MAGE-A3_ affitoxin 172-cpp compared with Z_MAGE-A3_ 172 (**Figure 3D**).

**Figure 3:**
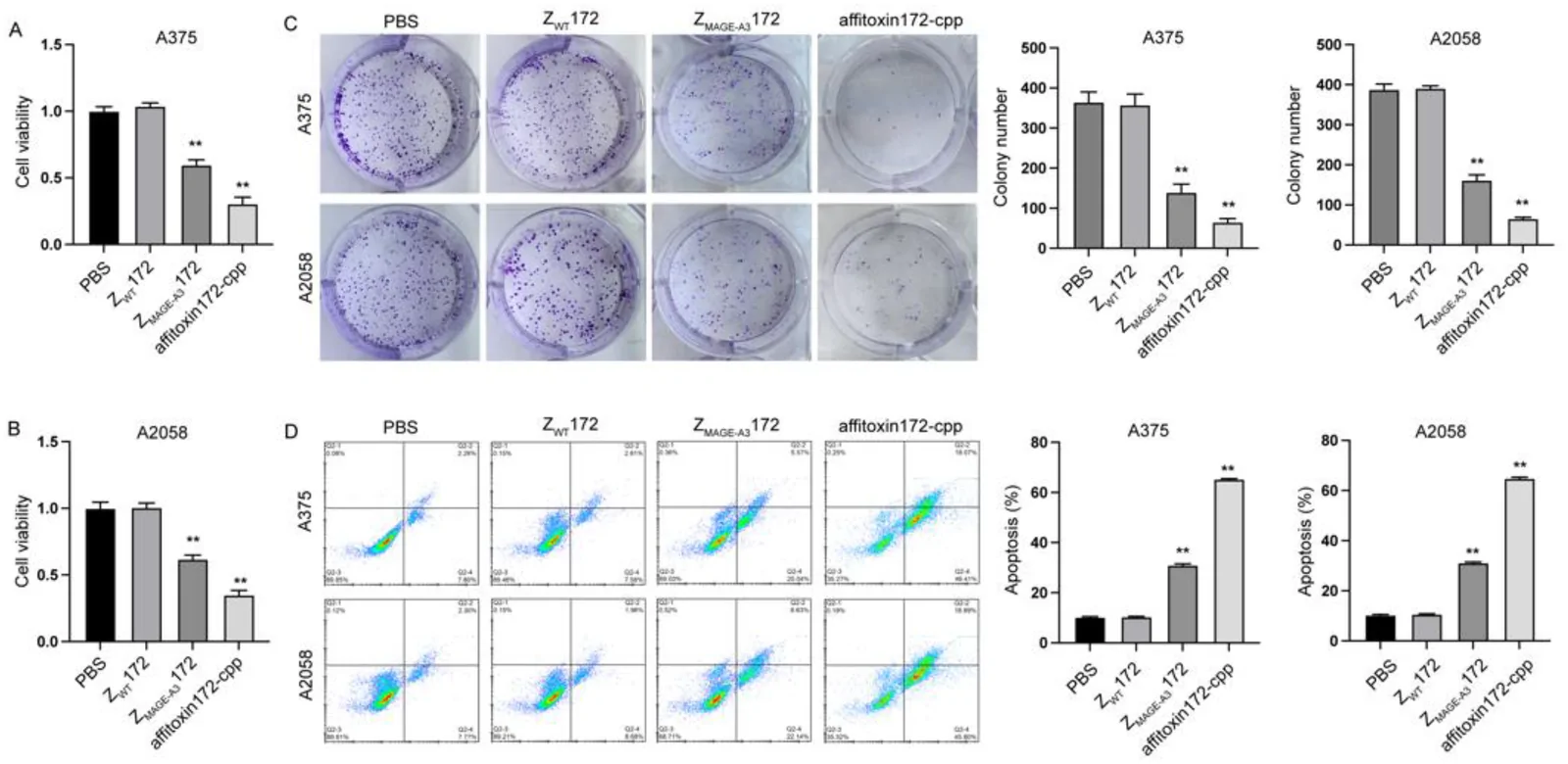
The ZMAGE-A3 affitoxin 172-cpp targets tumor cell proliferation *in vitro*. A375 and A2058 cells were treated with Z_wt_, Z_MAGE-A3_ affitoxin 172-cpp and Z_MAGE-A3_ 172, respectively. **(A)** Cell viability measured by CCK-8 assay. **(B)** Cell proliferation was detected by colony formation assay. **(C)** Cell apoptosis determined by flow cytometry. Data are presented as mean ± SD from three independent experiments. Statistical significance was determined by one-way ANOVA followed by Tukey’s HSD post-hoc test for comparisons among A375, A2058 and A431 groups. **P < 0.01; ns, not significant.

### The Z_MAGE-A3_ affitoxin 172-cpp targets tumors in nude mice *in vivo*

Next, we used NIR optical imaging to determine the tumor-targeting capacities of Z_wt_, Z_MAGE-A3_ affitoxin 172-cpp. Intravenous injections of DyLight755-labeled Z_wt_, Z_MAGE-A3_ affitoxin 172-cpp molecules were given to athymic nude mice harboring A375 and A2058 (MAGE-A3 positive) xenografts (**Figure 4A**) respectively. We found that high-contrast fluorescence signals of tumor sites occurred 1 hour after injection in both xenograft models, crested 12 h after injection, and persisted for 24 h after injection. We further investigated the *in vivo* anti-tumor effects of Z_MAGE-A3_ affitoxin 172-cpp using these two xenograft models. The treatment with Z_MAGE-A3–_172 significantly suppressed *in vivo* growth of A375 and A2058 tumors, exhibiting similar effects with anti-MAGE-172 antibody (**Figure 4A**). Noteworthy, Z_MAGE-A3_ affitoxin 172-cpp exhibited stronger anti-tumor ability compared with both the Z_MAGE-A3–_172 and anti-MAGE-172 antibody (**Figures 4B, C**).

**Figure 4:**
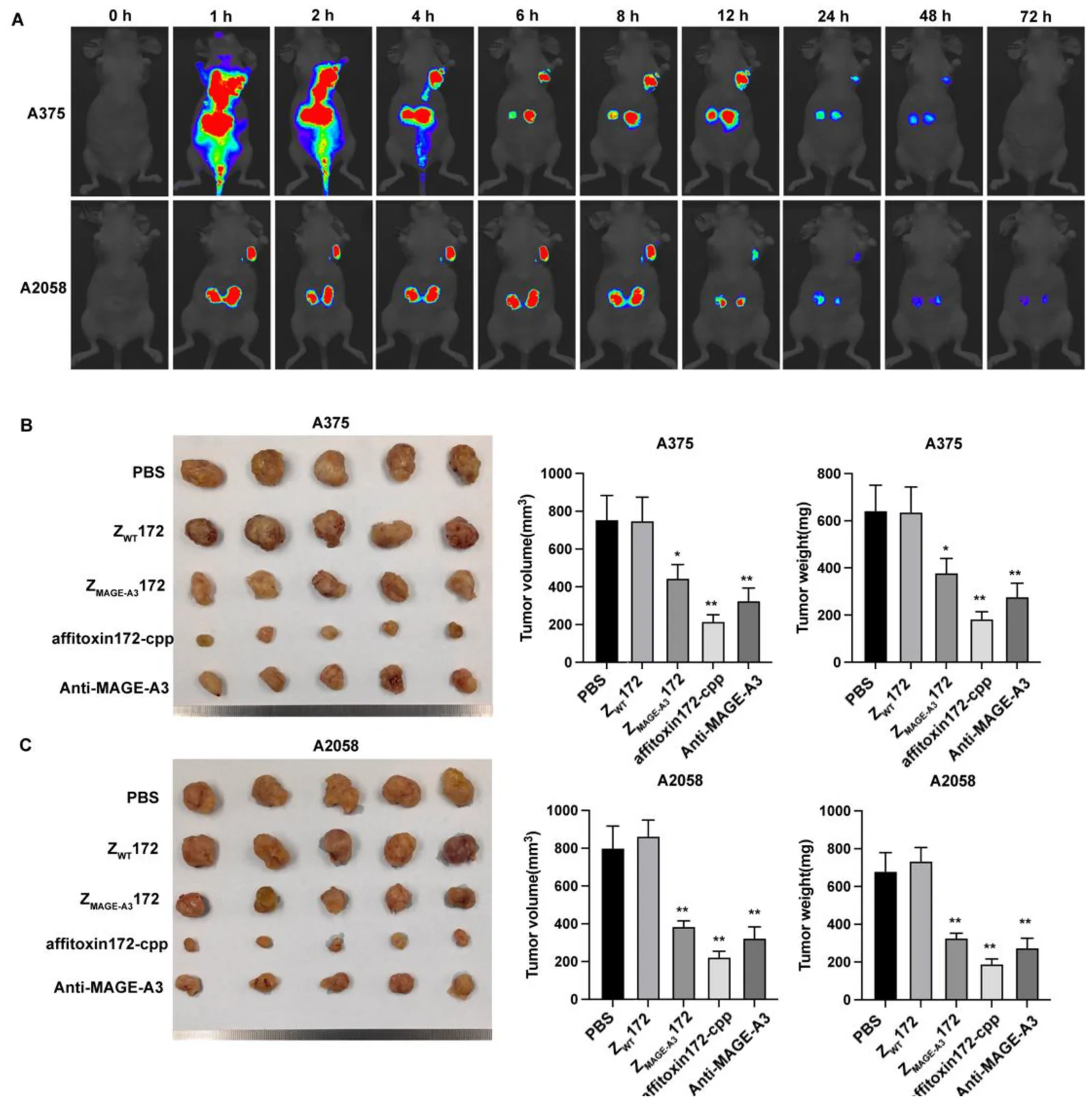
The ZMAGE-A3 affitoxin 172-cpp targets tumors in nude mice *in vivo*. **(A)** NIR image and Kidney/skin ratio of mice were performed at various time points after injection with DyLight755-labeled affibody. **(B, C)** Xenograft tumor size after treatment with Z_wt_, Z_MAGE-A3_ 172, Z_MAGE-A3_ affitoxin 172-cpp and anti-MAGE-172 antibody. Data are presented as mean ± SD from three independent experiments. Statistical significance was determined by one-way ANOVA followed by Tukey’s HSD post-hoc test for comparisons among A375, A2058 and A431 groups. *P < 0.05, **P < 0.01; ns, not significant.

## Discussion

Over hundreds of publications have documented over 43 affibody molecules targeting a variety of molecular targets, encompassing proteins and peptides. These targets include not only cell membrane proteins that are intimately linked to tumorigenesis, such as HER2, EGFR, and VEGF (12, 15, 16), but also key signaling molecules like H-Ras and c-Jun (17, 18). Additionally, the affibody molecules have been developed against viral proteins, including HPV 16/18 E7, Epstein-Barr virus LMP2, and the human immunodeficiency virus GP120 (19–21).

Affibody molecules offer several notable advantages over monoclonal antibodies in the realm of molecular imaging, making them highly appealing for cancer diagnostic research. Structurally, affibodies are considerably smaller than monoclonal antibodies, a distinction that carries significant practical benefits. Their compact size facilitates superior tissue penetration and swift targeting of tumor sites. Additionally, when compared to small-molecule probes, affibodies achieve an optimal balance between size and clearance, which enhances tissue targeting and imaging contrast. However, they also introduce unique pharmacokinetic challenges that warrant future investigation and attention. Furthermore, affibody molecules are recognized for their robustness and exceptional stability. In this study, we utilized the MAGE-A3 affibody to design an affitoxin for targeted therapy of tumors.

ZMAGE-A3 affitoxin 172-cpp is a conjugate comprising a carrier component, the MAGE-A3 affibody, and a toxin component, the PE38KDEL toxin, both linked to a cell-penetrating peptide (cpp). The PE38KDEL toxin has been engineered to enhance its specificity and toxicity by extending its residence in the endoplasmic reticulum (ER) through interaction with the KDEL receptor on the ER membrane (22). This toxin exerts its cytotoxic effect by inhibiting protein synthesis via ADP-ribosylation of elongation factor 2 (EF2), thereby initiating apoptosis (23, 24). The incorporation of the cpp into the affitoxin facilitates enhanced cellular internalization and toxin delivery, surmounting the hydrophobic barrier of cellular membranes (25–27). This modification is expected to augment the biological activity of the affitoxin and bolster its cell-targeting efficacy. Based on the results from *in vitro* cellular localization and *in vivo* imaging experiments, we have verified the enhanced cell internalization ability and tumor targeting ability. Furthermore, the results from *in vitro* experiments and xenograft tumor model indicated that ZMAGE-A3 affitoxin 172-cpp exhibited stronger anti-growth ability compared with ZMAGE-A3–172 and anti-MAGE-A3 antibody.

In the *in vivo* NIR imaging experiment (**Figure 4A**), we observed a notable difference in the early distribution patterns between A375 and A2058 xenografts. In A375 tumors, strong fluorescence signals were detected as early as 1 hour post-injection and became more confined to the tumor region, whereas in A2058 tumors, the signal at 1 hour was weaker and appeared more diffuse. This discrepancy may be attributed to several factors. First, the two cell lines may differ in their baseline MAGE-A3 expression levels; although both were confirmed positive by qPCR (**Figure 2A**), quantitative comparison suggested that A375 cells expressed higher MAGE-A3 mRNA levels than A2058 cells, which could lead to faster and stronger affitoxin binding. Second, differences in tumor vascularization and permeability might affect the initial extravasation and accumulation of the affitoxin (28). Third, variations in interstitial fluid pressure or stromal architecture between the two xenograft models could influence the diffusion kinetics (29, 30). Quantitative analysis of tumor-to-background ratios (TBR) at each time point would help clarify these possibilities (31); however, such analysis was not performed in the current study due to the lack of standardized region-of-interest measurements across all time points. Future studies incorporating dynamic contrast-enhanced imaging or ex vivo biodistribution assays are warranted to dissect the underlying mechanisms.

To evaluate the off-target specificity of ZMAGE-A3 affitoxin 172-cpp, we included A431 cells (MAGE-A3-negative) as controls in the qPCR and immunofluorescence experiments (**Figure 2**). No significant red fluorescence was observed in A431 cells after incubation with the affitoxin, indicating minimal non-specific binding. Nevertheless, a more comprehensive evaluation using additional MAGE-A3-negative cell lines and normal human tissues (e.g., primary fibroblasts or keratinocytes) would further strengthen the specificity claim. Importantly, affibody molecules are known to undergo rapid renal clearance, resulting in low accumulation in normal organs, which is favorable for reducing off-target toxicity (32). This is consistent with our observation that DyLight755-labeled affitoxin was largely cleared from the circulation by 48 hours post-injection (**Figure 4A**).

## Conclusion

To summarize, we have successfully engineered an innovative affitoxin, ZMAGE-A3 affitoxin 172-cpp, which exhibits high affinity and specificity for binding to MAGE-A3 in tumor cells. This affitoxin not only targets MAGE-A3-positive tumor cells but also effectively induces malignant cell death *in vitro* and *in vivo*. The MAGE-A3-targeting affitoxin presents itself as a highly promising therapeutic agent, capable of being utilized as a treatment to address efficient tumor therapy.

## Data Availability

The original contributions presented in the study are included in the article/supplementary material. Further inquiries can be directed to the corresponding author.
